# Two B-Box Proteins, GhBBX21 and GhBBX24, Antagonistically Modulate Anthocyanin Biosynthesis in *R1* Cotton

**DOI:** 10.3390/plants14152367

**Published:** 2025-08-01

**Authors:** Shuyan Li, Kunpeng Zhang, Chenxi Fu, Chaofeng Wu, Dongyun Zuo, Hailiang Cheng, Limin Lv, Haiyan Zhao, Jianshe Wang, Cuicui Wu, Xiaoyu Guo, Guoli Song

**Affiliations:** 1 Anyang Institute of Technology, Anyang 455000, China; zhangkunpengag@163.com (K.Z.); 19711098083@163.com (C.F.); 20160425@ayit.edu.cn (C.W.); zhaohaiyan111111@163.com (H.Z.); wongjaction@163.com (J.W.); guoxiaoyu0223@163.com (X.G.); 2 Henan Provincial Engineering Technology Research Center for Comprehensive Utilization of Medicinal Plants, Anyang 455000, China; 3 Institute of Cotton Research of Chinese Academy of Agricultural Science, Anyang 455000, China; zdy041@163.com (D.Z.); pser2010@163.com (H.C.); llm0372@126.com (L.L.); 4 Cotton Research Institute, Shanxi Agricultural University, Yuncheng 044000, China; wucuicui19821021@126.com

**Keywords:** anthocyanin biosynthesis, BBX proteins, cotton

## Abstract

The red plant phenotype of *R1* cotton is a genetic marker produced by light-induced anthocyanin accumulation. *GhPAP1D* controls this trait. There are two 228 bp tandem repeats upstream of *GhPAP1D* in *R1* cotton. In this study, GUS staining assays in transgenic *Arabidopsis thaliana* (L.) Heynh. demonstrated that tandem repeats in the *GhPAP1D* promoter-enhanced transcriptional activity. *GhPAP1D* is a homolog of *A. thaliana AtPAP1*. *AtPAP1’s* expression is regulated by photomorphogenesis-related transcription factors such as AtHY5 and AtBBXs. We identified the homologs of *A. thaliana AtHY5*, *AtBBX21*, and *AtBBX24* in *R1* cotton, designated as *GhHY5*, *GhBBX21*, and *GhBBX24*, respectively. Y1H assays confirmed that GhHY5, GhBBX21, and GhBBX24 each bound to the *GhPAP1D* promoter. Dual-luciferase reporter assays revealed that GhHY5 weakly activated the promoter activity of *GhPAP1D*. Heterologous expression assays in *A. thaliana* indicated that *GhBBX21* promoted anthocyanin accumulation, whereas *GhBBX24* had the opposite effect. Dual-luciferase assays showed GhBBX21 activated *GhPAP1D* transcription, while GhBBX24 repressed it. Further study indicated that GhHY5 did not enhance GhBBX21-mediated transcriptional activation of *GhPAP1D* but alleviates GhBBX24-induced repression. Together, our results demonstrate that GhBBX21 and GhBBX24 antagonistically regulate anthocyanin accumulation in *R*1 cotton under GhHY5 mediation, providing insights into light-responsive anthocyanin biosynthesis in cotton.

## 1. Introduction

Anthocyanins are a class of water-soluble pigments that provide vibrant and diverse colors to flowers, fruits, leaves, and other tissues [1]. Anthocyanins play important roles in protecting plants from biotic and abiotic stresses, such as pathogen attack, extreme temperature, and ultraviolet radiation [2,3]. The biosynthesis of anthocyanins is through the phenylpropanoid pathway, which includes a series of enzymes encoded by structural genes. These structural genes are transcriptionally regulated by MYB/bHLH/WD40 (MBW) protein complex. The MBW complex promotes anthocyanin biosynthesis by binding to structural gene promoters and activating their transcription [4].

Light exposure can induce the accumulation of anthocyanins in plants. The basic leucine zipper (bZIP) protein ELONGATED HYPOCOTYL (HY5) plays a critical role in photomorphogenesis, regulating hypocotyl elongation, anthocyanin, and chlorophyll accumulation, and integrating multiple external and internal signaling pathways [5]. HY5 can bind to cis-elements such as ACE box (ACGT), T/G-box (CACGTT), C-box (GTCANN), and E-box (CAATTG) in the promoters of many genes involved in anthocyanin synthesis. In *Arabidopsis thaliana* (L.) Heynh., AtHY5 directly targets the G-box and ACE box in the *AtPAP1* promoter [6]. In *Malus* × *domestica* Borkh., MdHY5 binds to the E-box and G-box in the *MdMYB10* promoter [7]. In red *Pyrus pyrifolia*, PyHY5 specifically recognizes the G-box within the promoters of *PyWD40* and *PyMYB10* [8]. Early studies have shown that overexpression of *AtHY5* did not lead to the expected strong photomorphogenic phenotype. The analysis of the protein structure of AtHY5 indicates that it lacks a transcriptional activation domain. In recent years, an increasing number of experimental results have shown that the binding of HY5 to the promoter of target genes is constitutive, requiring cooperation with other cofactors to activate downstream gene expression [9,10].

B-Box (BBX) proteins are zinc-finger transcription factors with one or two BBX domains and sometimes a CCT domain [11]. Group IV B-box proteins act as critical HY5 cofactors, playing essential roles in regulating anthocyanin accumulation in plants. In *A. thaliana*, AtBBX20-AtBBX23 positively regulate anthocyanin accumulation, whereas AtBBX24 and AtBBX25 negatively regulate this process [9]. In *M*. × *domestica*, MdBBX22 forms a functional dimer with MdHY5, which directly binds to the promoters of *MdMYB10* and *MdCHS* to activate their expression, ultimately enhancing anthocyanin biosynthesis [10]. MdBBX37 binds to the key transcription factors MdMYB1 and MdMYB9 in the anthocyanin pathway, thereby suppressing anthocyanin biosynthesis [12]. In *P. pyrifolia*, PpBBX18 interacts with PpHY5 to transcriptionally activate *PpMYB10*, which drives anthocyanin biosynthesis. PpBBX21 interacts independently with PpHY5 and PpBBX18, preventing the formation of the PpHY5-PpBBX18 dimer and thereby suppressing anthocyanin biosynthesis [13].

*Gossypium hirsutum* L. is one of the world’s most widely cultivated and economically important cash crops. The red plant (*R1*) gene is a traditional genetic marker in *G. hirsutum*. The red color of *R1 G. hirsutum (R1 cotton)* is produced by the anthocyanin accumulation and distributed throughout the stems and leaves, making the entire plant red. The *R1* gene is an incomplete dominant single gene. Genetic mapping and transgenic *G. hirsutum* phenotype analysis of the recombinant inbred line (RIL) populations of T586 (red plant, *R1* cotton) × Yumian1 (green leaf, *GL* cotton) confirmed that *R1* was a key MYB transcription factor involved in the regulation of anthocyanin biosynthesis, namely *GhPAP1D*. The *GhPAP1D* gene was rapidly upregulated under light exposure in *R1* cotton. Further research showed there were two 228 bp tandem repeats upstream of *GhPAP1D* in *R1* cotton [2,14]. However, the mechanism by which tandem repeats in the *GhPAP1D* promoter mediate light-induced transcriptional upregulation remains unclear. This study aimed to identify transcription factors responsible for light-induced regulation of *GhPAP1D*. Our findings demonstrate that two B-box transcription factors, GhBBX21 and GhBBX24, antagonistically regulate anthocyanin biosynthesis in *R1* cotton.

## 2. Results

### 2.1. Comparative Analysis of the Upstream Promoter Regions of GhPAP1D Between GL and R1 Cotton

**Figure 1:**
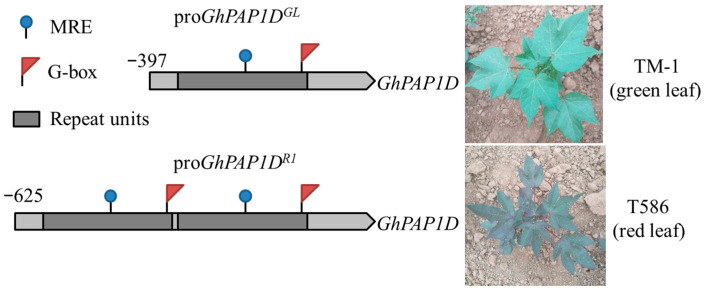
Schematic diagram of structural differences between the *GhPAP1D* promoters in *GL* and *R1* cotton lines. The pro*GhPAP1D^GL^* (TM-1, *GL* cotton) and proGhPAP1D^R1^ (T586, *R1* cotton) sequences are identical except for the copy number of a 228 bp tandem repeat: one copy in *GL* vs. two copies in *R1*. The diagram also indicates cis-acting elements (MRE: TCTCTTA; G-box: CACGTC).

To investigate the regulatory elements within the upstream promoter region of *GhPAP1D*, we cloned and compared the promoter sequences from both *GL* and *R1* cottons (Supplementary Table S1). In *GL* cotton, a 404 bp PCR product (pro*GhPAP1D^GL^*) was amplified as expected, while in *R1* cotton, the length of the PCR product was 632 bp (pro*GhPAP1D^GL^*). The sequences are identical except for the number of 228 bp tandem repeats (one in *GL* cotton, two in *R1* cotton). The results are consistent with a previous report [14]. The Plantcare website [15] and the published literature [16] were used to predict the cis-acting elements of pro*GhPAP1D^GL^* and pro*GhPAP1D^R1^*. Compared with pro*GhPAP1D^GL^*, the 228 bp insertion of pro*GhPAP1D^R1^* adds a MYB binding element MRE (TCTCTTA) and a photoresponsive element G-box (CACGTC) (Figure 1).

### 2.2. Activity Analysis of proGhPAP1DGL and proGhPAP1DR1 in Response to Light

To investigate whether tandem repeated insertion enhances the promoter activity of *GhPAP1D* in *R1* cotton, the promoters (pro*GhPAP1D^GL^* and pro*GhPAP1D^R1^*) were fused to the GUS reporter gene and introduced into *A. thaliana* through *Agrobacterium*-mediated gene transfer, with 35S:*GUS* serving as a positive control. The 7-day-old T_3_ *A. thaliana* transgenic seedlings containing the homozygous recombinant transgene were cultured under normal light (16 h light/8 h dark) or continuous darkness (24 h dark) and then subjected to histochemical staining for GUS activity.

**Figure 2:**
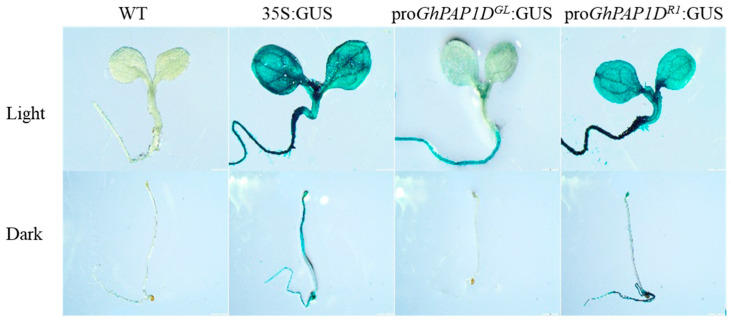
Histochemical staining of GUS activity in 7-day-old seedlings of pro*GhPAP1D^GL^:GUS* and pro*GhPAP1D^R1^:GUS* transgenic lines under normal light (16 h light/8 h dark) or continuous darkness (24 h dark). In seedlings grown under normal light, GUS staining intensity was significantly stronger in pro*GhPAP1D^R1^:GUS lines* than in pro*GhPAP1D^GL^:GUS* lines. pro*GhPAP1D^R1^:GUS* seedlings grown under continuous darkness exhibited weaker GUS staining than those grown under normal light.

The results are listed in Figure 2. In seedlings grown under normal light, the GUS-staining intensity in pro*GhPAP1D^R1^:GUS* transgenic lines was much deeper than in pro*GhPAP1D^GL^:GUS* lines, indicating that tandem repeated insertion enhanced the promoter activity of *GhPAP1D* in *R1* cotton. Furthermore, pro*GhPAP1D^R1^:GUS* seedlings grown in continuous darkness exhibited weaker GUS staining than those grown in normal light, demonstrating that *GhPAP1D* expression in *R1* cotton is light-responsive.

### 2.3. Autoregulation of GhPAP1D in the Dual-Luciferase Transient Tobacco Assays

In red-fleshed *M*. × *domestica*, the multiple repeats in the promoter of *MdMYB10* create a positive feedback loop (autoregulation), resulting in elevated *MdMYB10* transcript levels and subsequent anthocyanin accumulation in *M*. × *domestica* flesh [17]. In *R1* cotton, the repeated sequence of pro*GhPAP1D^R1^* adds a MYB binding element MRE (TCTCTTA). To investigate whether *GhPAP1D* exhibits autoregulation, dual-luciferase assays were used to quantify the activity of the two types of the *GhPAP1D* promoter in *GL* and *R1* cottons. The transactivation activities of pro*GhPAP1D^GL^* and pro*GhPAP1D^R1^* were assessed by fusing them to *LUC* and measuring LUC luminescence relative to 35S:*REN* after transient expression in *Nicotiana. benthamiana* leaves. As shown in Supplementary Figure S1, the co-infiltration of 35S:*GhPAP1D* with pro*GhPAP1D^GL^*:*LUC* increased transactivation activity by 1.7-fold (1.7031 ± SE 0.1901), while 35S:*GhPAP1D* transactivated pro*GhPAP1D^R1^*:*LUC* 2-fold (2.0671 ± SE 0.13027) compared to the background. The above results indicate that *GhPAP1D* exhibits autoregulatory activity in both *GL* and *R1* cottons. However, no significant difference was observed in the effect of 35S:*GhPAP1D* on pro*GhPAP1D^GL^* and pro*GhPAP1D^R1^*, suggesting that autoregulation is not the primary factor driving the higher expression of *GhPAP1D* in *R1* cotton compared to *GL* cotton.

### 2.4. Identification of GhHY5 in R1 Cotton

It is known that HY5 plays an important role in integrating the light signals with pigment accumulation. HY5 was identified to bind directly to the promoters of its target genes on G-box, ACE-box, or E-Box [6,7]. The repeated sequence of pro*GhPAP1D^R1^* adds a HY5-binding element G-box (CACGTC). To examine the function of HY5, the HY5 sequence in *R1* cotton was cloned and analyzed. The HY5 in *R1* cotton had a 510 bp ORF encoding a protein containing 169 amino acids and was named *GhHY5*. The deduced amino acid sequence of GhHY5 had 74.71% sequence identity with AtHY5, and the secondary structure of the GhHY5 protein had a bZIP domain (amino acid residues 92–142) on the C-terminal side (Supplementary Figure S2).

### 2.5. Identification of BBX Subfamily IV Gene Family from Gossypium hirsutum *L.*

**Figure 3:**
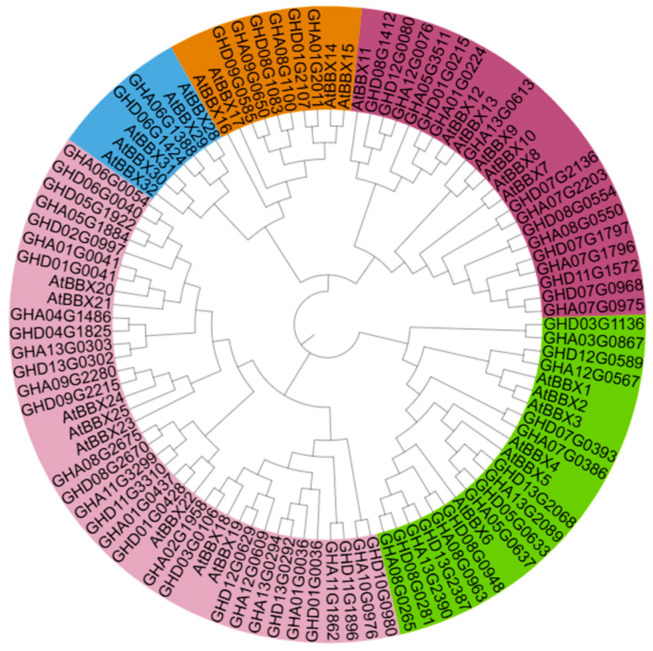
Phylogenetic tree of BBX gene family from *G. hirsutum* and *A. thaliana* (note: the green color region indicates Subgroup I, the purple color region indicates Subgroup II, the brown color indicates Subgroup III, the pink color indicates Subgroup IV, and the blue color indicates Subgroup V).

Members of the B-Box (BBX) subfamily IV act as transcriptional cofactors of HY5, participating in the regulation of anthocyanin biosynthesis across various plant species, such as *A. thaliana*, *M*. × *domestica*, *P. pyrifolia*, and *Prunus avium*. Thirty-two protein sequences of the BBX family from *A. thaliana* were downloaded from the TAIR database. Seventy-one protein sequences of the BBX family from *G. hirsutum* were retrieved from the CottonFGD database. We constructed a phylogenetic tree of the BBX family between *A. thaliana* and *G. hirsutum* (Figure 3). In *G. hirsutum*, 31 protein sequences of the BBX family were clustered into subfamily IV. It can be seen that the genes of Group IV have multiple copies in *G. hirsutum*, suggesting that these genes may result in functional redundancy.

The results of protein sequence alignment and structural analysis for subfamily IV of the BBX family in *A. thaliana* and *G. hirsutum* are presented in Supplementary Figure S3. The N-terminal amino acids containing two B-box domains have high homology, and the B-box domain is considered a key region for binding to the bZIP domain of HY5 [18,19]. After the second B-box domain, the amino acid residue consistency is very low, which is a key region for functional differences in the IV subfamily members [20]. The amino acid sequences of AtBBX20, AtBBX21, and their *G. hirsutum* homologous proteins all contain a TAD domain, which is considered a key region for initiating downstream gene transcription [9,18,21].

To examine the function of *GhBBXs*, the alleles of *GH_A05G1884* (the homolog gene of *AtBBX21*) and *GH_A09G2280* (the homolog gene of *AtBBX24*) were cloned from the *R1* plant, and were named *GhBBX21* and *GhBBX24*, respectively.

### 2.6. GhHY5, GhBBX21, and GhBBX24 Are Transcriptional Activators Localized in the Nuclei

**Figure 4:**
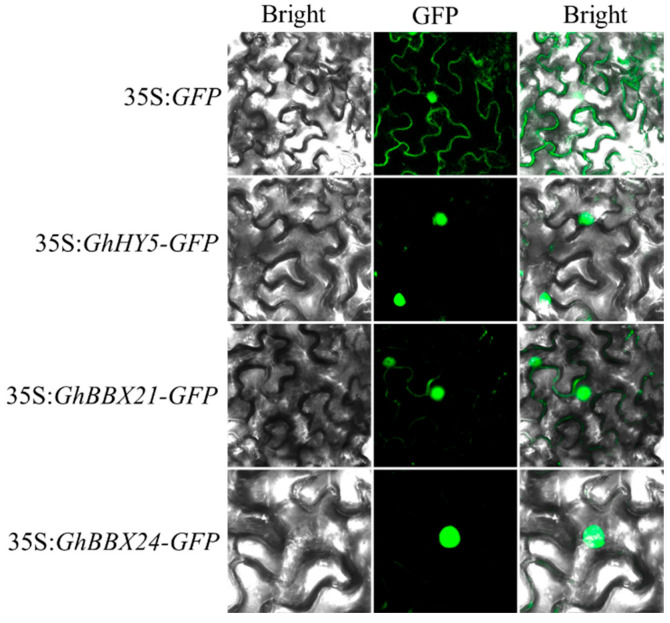
Subcellular localization of GhHY5-GFP, GhBBX21-GFP, and GhBBX24-GFP in *N. benthamiana* leaf epidermal cells.

To determine the subcellular localization of the GhHY5, GhBBX21, and GhBBX24, *N. benthamiana* leaves were separately transfected with 35S:*GFP*, 35S:*GhHY5-GFP*, 35S:*GhBBX21-GFP*, and 35S:*GhBBX24-GFP* constructs. As shown in Figure 4, the 35S:*GFP* fusion protein exhibited diffuse localization in both the nucleus and cytoplasm, whereas the 35S:*GhHY5-GFP*, 35S:*GhBBX21-GFP*, and 35S:*GhBBX24-GFP* fusion proteins displayed exclusive nuclear localization. These findings confirm that GhHY5, GhBBX21, and GhBBX24 function as nuclear-localized transcriptional activators.

### 2.7. GhBBX21 and GhBBX24 Antagonistically Modulate Anthocyanin Accumulation in Transgenic A. thaliana Seedlings

**Figure 5:**
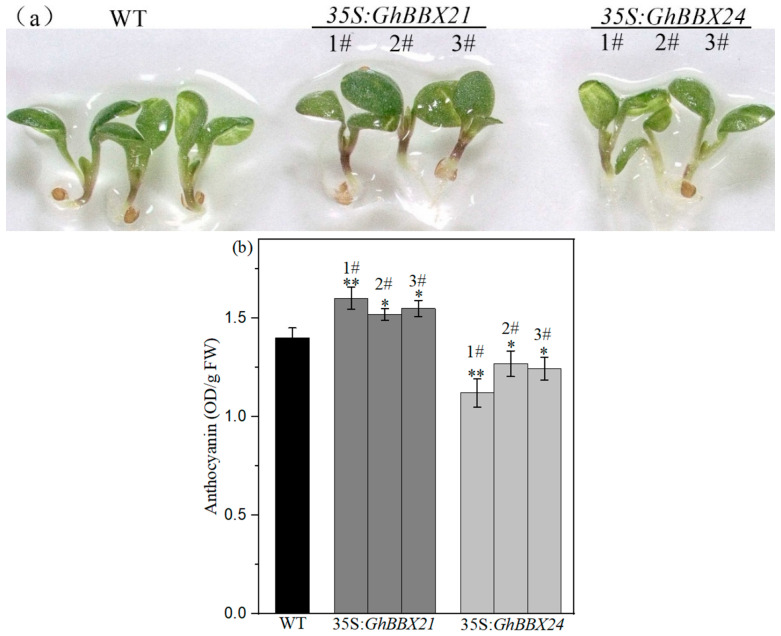
Functional analysis of *GhBBX21* and *GhBBX24* in *A. thaliana*. (**a**) Phenotypes of positive transgenic lines. Three independent transgenic lines were used for each gene. (**b**) Anthocyanin contents in seedlings of positive transgenic lines. FW: fresh weight. Error bars represent the mean ± SE of three biological replicates. Statistical significance was analyzed using Student’s *t*-test (* *p* < 0.05, ** *p* < 0.01, vs. WT control).

To functionally characterize *GhBBX21* and *GhBBX24*, we generated stable transgenic *A. thaliana* (WT) lines heterologously overexpressing them. For each construct, more than three independent overexpression lines were established. Compared with wild-type controls, 35S:*GhBBX21* transgenic lines showed increased anthocyanin accumulation, while 35S:*GhBBX24* lines exhibited reduced accumulation (Figure 5a,b). The above results indicate that *GhBBX21* and *GhBBX24* antagonistically modulate anthocyanin accumulation in transgenic *A. thaliana* seedlings.

### 2.8. The Regulation of GhPAP1D by GhHY5, GhBBX21, and GhBBX24

**Figure 6:**
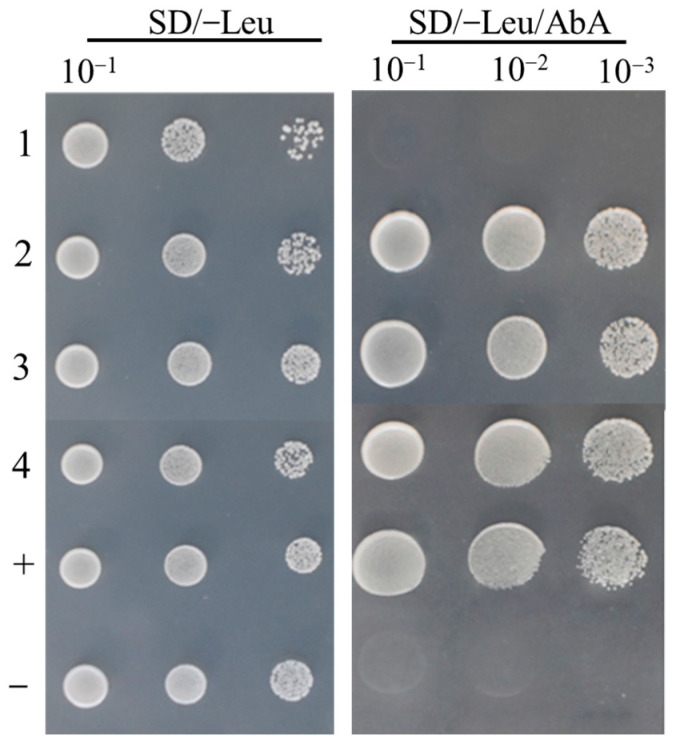
Yeast one-hybrid assay detecting binding of GhHY5, GhBBX21, and GhBBX24 to pro*GhPAP1D^R1^*. Transformants were grown on SD/-Ura/-Leu medium supplemented with 200 ng/mL aureobasidin A. Lanes: (1) pGADT7 + pAbAi- pro*GhPAP1D^R1^* (empty vector control); (2) pGADT7-GhBBX21 + pro*GhPAP1D^R1^*; (3) pGADT7-GhBBX24 + pro*GhPAP1D^R1^*; (4) pGADT7-GhHY5 + pro*GhPAP1D^R1^*; (+) pGADT7-rec53 + pAbAi-p53 (positive control); (−) pGADT7 + pAbAi-p53 (negative control).

**Figure 7:**
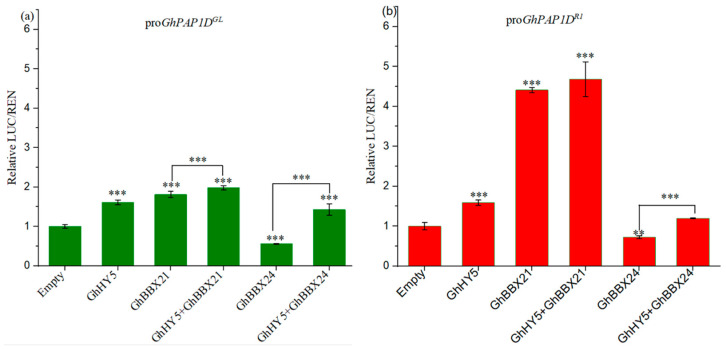
GhBBX21, GhBBX24, and GhHY5 activated transcription of downstream genes of pro*GhPAP1D^GL^* and pro*GhPAP1D^R1^* in dual-luciferase assays. (**a**) Reporter constructs: pro*GhPAP1D^GL^*-pGreen0800-LUC. (**b**) Reporter constructs: pro*GhPAP1^R1^*-pGreen0800-LUC. For both assays, the effector constructs (from left to right) were empty (pGreenII 62-SK), GhHY5 (GhHY5-pGreenII 62-SK), GhBBX21 (GhBBX21-pGreenII 62-SK), GhHY5 + GhBBX21 (GhHY5-pGreenII 62-SK + GhBBX21-pGreenII 62-SK), GhBBX24 (GhBBX24-pGreenII 62-SK), and GhHY5 + GhBBX24 (GhHY5-pGreenII 62-SK + GhBBX24-pGreenII 62-SK). *Firefly luciferase* activity was normalized to *Renilla luciferase* activity. Error bars represent the mean ± SE of four biological replicates. Significant differences were determined by Student’s *t*-test (* *p* < 0.05, ** *p* < 0.01, *** *p* < 0.001, vs. empty vector control). Significant difference (underlined) between GhBBX21/GhBBX24 + GhHY5 co-expression and GhBBX21/GhBBX24 alone.

To verify whether GhHY5, GhBBX21, and GhBBX24 are involved in regulating the transcription of *GhPAP1D* in *R1* cotton, we employed Y1H assays to validate their direct binding to the promoter of *GhPAP1D*. As shown in Figure 6, the pro*GhPAP1D^R1^*-pAbAi bait yeast cells did not grow on selective medium supplemented with aureobasidin A (AbA, 200 ng/mL). However, when co-transformed with GhHY5, GhBBX21, or GhBBX24, the pro*GhPAP1D^R1^*-pAbAi bait yeast cells could grow on the same selective medium. This confirms the interaction of GhHY5, GhBBX21, or GhBBX24 with pro*GhPAP1D^R1^*. Furthermore, we detected the activity of GhHY5, GhBBX21, and GhBBX24 on downstream genes of pro*GhPAP1D^GL^* and pro*GhPAP1D^R1^* through dual-luciferase assay. As shown in Figure 7, GhHY5 weakly activated downstream gene transcription of pro*GhPAP1D^R1^* (1.8-fold higher than the control), and there was basically no activation effect on downstream gene transcription of pro*GhPAP1D^GL^*. GhBBX21 had weak activation effects on downstream genes of pro*GhPAP1D^GL^*, which was 2.0-fold higher than the empty control, while it had significant activation effects on downstream genes of pro*GhPAP1D^R1^*, which was 4.5-fold higher than the control. Compared with the presence of GhBBX21 alone, the co-injection of GhBBX21 and GhHY5 does not significantly enhance the expression of downstream genes pro*GhPAP1D^GL^* and pro*GhPAP1D^R1^*. GhBBX24 had a negative regulatory effect on downstream genes of pro*GhPAP1D^GL^* and pro*GhPAP1D^R1^*, but when co-existing with GhHY5, this negative regulatory effect was inhibited. From the above assays, it can be concluded that GhHY5 alone is insufficient to activate the transcription of *GhPAP1D* in both *R1* and *GL* cotton, whereas GhBBX21 significantly enhances the transcription of *GhPAP1D* in *R1* cotton. Notably, the tandem repeats within the *GhPAP1D* promoter in *R1* cotton evidently amplify promoter activity. In contrast to GhBBX21, which positively regulates *GhPAP1D* expression, GhBBX24 negatively controls *GhPAP1D* synthesis.

### 2.9. GhBBX21 and GhBBX24 Proteins Interact with GhHY5

**Figure 8:**
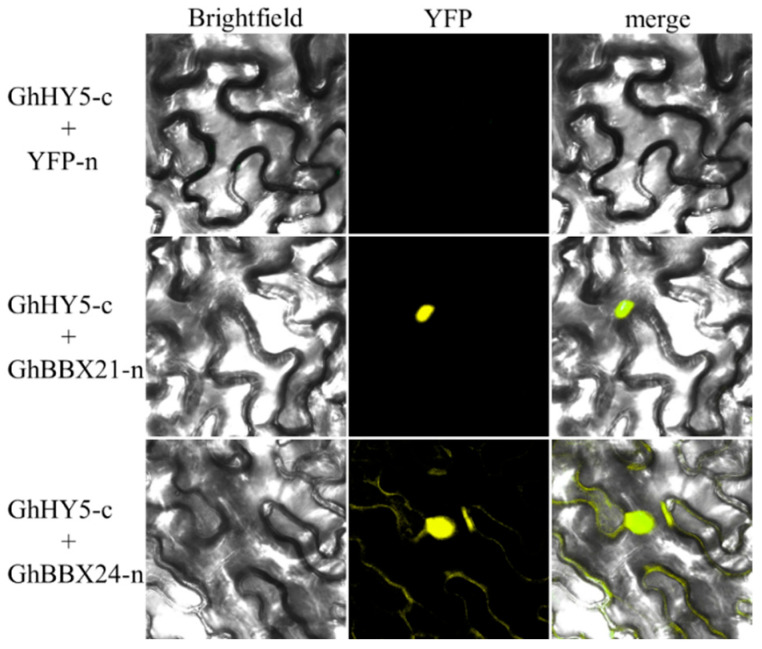
Interaction between GhBBX21/GhBBX24 with GhHY5 identified using bimolecular fluorescence complementation assay in *N. benthamiana* leaf epidermal cells.

While many BBX proteins are known to regulate anthocyanin biosynthesis through HY5-dependent pathways in various plant species, we investigated whether GhBBX21 and GhBBX24 physically interact with GhHY5 using BiFC assays in *N. benthamiana* leaves. As shown in Figure 8, YFP fluorescence signals were observed in the nuclei of epidermal cells when 35S: *GhHY5-c* was co-expressed with either 35S:*GhBBX21-n* or 35S:*GhBBX24-n*. In contrast, no fluorescence was detected in any negative control combinations. These results demonstrate that both GhBBX21 and GhBBX24 can directly interact with GhHY5.

## 3. Discussion

### 3.1. Tandem Repeats in the Promoter Region Enhance Light-Responsive Transcription of Ghpap1d

Light is a critical environmental regulator of anthocyanin biosynthesis [22,23]. In many plants, anthocyanin synthesis requires light induction. Evidence indicates that UV-B and blue light are key regulators of anthocyanin accumulation in plants. These light spectra induce the expression of key transcription factors (e.g., MYB, BBX, and HY5), which subsequently activate structural genes associated with anthocyanin biosynthesis through direct or indirect regulatory mechanisms [24,25,26].

The red plant phenotype of *R1* cotton is light-dependent. Under natural sunlight, the phenotype is clearly visible, but anthocyanin accumulation decreases significantly when plants are grown in a glasshouse or shade. This phenotype is caused by two 228 bp tandem repeats in the *GhPAP1D* promoter in *R1* cotton, compared to just one in *GL* cotton [2,27]. In plants, cases of repeat insertions leading to increased promoter activity have been reported [17,28,29,30,31]. GUS staining of T_3_ transgenic *A. thaliana* seedlings demonstrated that pro*GhPAP1D^R1^* drives significantly higher transcriptional activation under light compared to pro*GhPAP1D^GL^*. These results indicate that the 228 bp repeat insertion enhances *GhPAP1D* promoter activity in *R1* cotton. The inserted repeat sequence contains both an MRE (MYB recognition element; TCTCTTA) and a light-responsive G-box (CACGTC), which may serve as binding sites for trans-acting factors that upregulate *GhPAP1D* expression.

### 3.2. Autoregulation Is Not the Main Driver of the Increased Expression of GhPAP1D in R1 Cotton

In red-fleshed *M*. × *domestica*, the multiple sequences in the *MdMYB10* promoter contain MYB binding elements, resulting in strong self-activation of *MdMYB10* [17]. To determine whether *GhPAP1D*’s elevated expression in *R1* cotton results from autoregulation, we compared the autoregulatory activity of pro*GhPAP1D^GL^* and pro*GhPAP1D^R1^* using dual-luciferase assays. While *GhPAP1D* shows autoregulatory activity in both *GL* and *R1* cotton, the similar activity between pro*GhPAP1D^GL^* and pro*GhPAP1D^R1^* indicates autoregulation is not the main driver of the increased expression of *GhPAP1D* in *R1* cotton.

### 3.3. GhHY5 Binds Constitutively to GhPAP1D Promoter

GUS staining assays indicate that pro*GhPAP1D^R1^* is induced by light. The G-box in the promoter is a key light-responsive element. In many plants, the bZIP transcription factor HY5, a key regulator of pigment accumulation, binds to G-box elements in anthocyanin biosynthetic gene promoters [8,9,26]. In this study, we cloned *GhHY5* from *R1* cotton and found its deduced protein sequence shares 74.12% identity with AtHY5. Y1H assays confirmed that GhHY5 binds to pro*GhPAP1D^R1^*. Dual-luciferase assays further revealed that GhHY5 activates downstream gene expression from pro*GhPAP1D^R1^* at 2-fold the control level, while its effect on pro*GhPAP1D^GL^* was negligible. These results suggest that GhHY5 alone cannot fully account for the high *GhPAP1D* expression in *R1* cotton. Like in other plants, GhHY5 may bind constitutively to the *GhPAP1D* promoter, and precise transcriptional regulation requires additional cofactors.

### 3.4. GhBBX21 and GhBBX24 Antagonistically Regulate GhPAP1D Expression, with GhBBX21 Significantly Activating the GhPAP1D Transcription in R1 Cotton

B-box subfamily IV members have been identified as regulators of anthocyanin biosynthesis in plants, with some B-box proteins positively regulating anthocyanin accumulation while others function as repressors [9,12,20,25,32,33,34]. Given the potential role of BBX subfamily IV in regulating anthocyanin accumulation in *R1* cotton, we constructed a phylogenetic tree of BBX proteins from *A. thaliana* and *G. hirsutum*. 31 BBX proteins in *G. hirsutum* were classified into subfamily IV, representing a significant expansion compared to only eight members in *A. thaliana*. This suggests that the subfamily IV genes may have important functional roles in *G. hirsutum*. We cloned and characterized the *A. thaliana AtBBX21/AtBBX24* orthologs from *R1* cotton, designated as *GhBBX21* and *GhBBX24*. Both GhBBX21 and GhBBX24 possess two conserved B-box domains. However, GhBBX21 additionally possesses a TAD, which is absent in GhBBX24. The B-Box domains are recognized as HY5-interaction motifs, while the TAD functions as a transcriptional activation domain. Heterologous expression in stable transgenic *A. thaliana* lines revealed that *GhBBX21* promoted anthocyanin accumulation, while *GhBBX24* functioned as a repressor. Y1H assays demonstrated that both GhBBX21 and GhBBX24 directly bound to the *GhPAP1D* promoter in *R1* cotton. The dual-luciferase assays showed that GhBBX21 initiated a high expression of pro*GhPAP1D^R1^* downstream genes, while having little effect on the transcriptional activity of pro*GhPAP1D^GL^* downstream genes. These results further indicated that the presence of tandem repeats in pro*GhPAP1D^R1^* enhanced the transcriptional activity of *GhPAP1D*. The dual-luciferase assays showed that GhBBX24 had a negative regulatory effect on *GhPAP1D*. The above results indicate that GhBBX21 and GhBBX24 antagonistically regulate *GhPAP1D* expression, with GhBBX21 significantly activating *GhPAP1D* transcription in *R1* cotton.

### 3.5. GhHY5 Interacts with Either GhBBX21 or GhBBX24 to Coregulate GhPAP1D Expression in R1 Cotton

B-box subfamily IV members are key cofactors in HY5-dependent photomorphogenesis. The regulatory mechanisms of BBX proteins and HY5 in anthocyanin synthesis are complex. In *A. thaliana*, AtBBX22 promotes anthocyanin accumulation. AtHY5 activates *AtBBX22* transcription by binding to its promoter, whereas AtBBX24 and AtBBX25 suppress *AtBBX22* expression by directly interacting with AtHY5 [19]. In *M*. × *domestica*, MdBBX22 forms a heterodimer with MdHY5 to activate *MdMYB10* and *MdCHS* expression, whereas MdBBX37 inhibits anthocyanin synthesis by repressing *MdHY5* transcription and interfering with key regulators MdMYB1 and MdMYB9 [12]. In grape hyacinth, MaBBX21 interacts with MaHY5 to activate *MaMybA* and *MaDFR* expression, whereas MaBBX51 disrupts the MaBBX20-MaHY5 complex and represses their transcription [35]. Notably, some studies report HY5-independent anthocyanin promotion by BBX proteins. In *P. avium*, PavBBX6 and PavBBX9 directly bind to G-box elements in the *PavUFGT* promoter to enhance light-induced anthocyanin synthesis [36]. In *Mangifera indica* L., MiBBX24 and MiBBX27 transcriptionally activate *MiMYB1* by binding to its promoter, thereby promoting anthocyanin accumulation in the fruit peel [24]. In our study, Y1H assays demonstrated that both GhBBX21 and GhBBX24 directly bound to the *GhPAP1D* promoter. BiFC assays showed that GhHY5 could interact with GhBBX21 and GhBBX24, respectively. Dual-luciferase assays indicated that GhHY5 did not significantly influence GhBBX21’s regulation of *GhPAP1D* transcription, and GhBBX21 itself significantly activated the transcription of *GhPAP1D* in *R1* cotton. Intriguingly, co-expression with GhHY5 abolishes GhBBX24-mediated repression of *GhPAP1D* transcription. Based on the structural analysis of GhBBX21 and GhBBX24 described earlier, we observe that GhBBX21 contains a TAD (transcriptional activation domain) that enables itself to activate downstream gene expression. In contrast, GhBBX24 lacks this domain. We propose that GhBBX24 represses *GhPAP1D* by competitively blocking transcriptional activators at the promoter. When GhHY5 interacts with GhBBX24, it facilitates the dissociation of GhBBX24 from the *GhPAP1D* promoter, thereby allowing other activators to bind and promote transcription.

## 4. Materials and Methods

### 4.1. Plant Materials and Growth Conditions

The *R1* cotton (*G. hirsutum* cv. T586) and *GL* cotton (*G. hirsutum* cv. TM-1) used for DNA extraction were cultivated in the experimental farm of Institute of cotton research, Chinese Academy of Agricultural Sciences, Anyang, Henan Province, China. *A. thaliana* seeds were sterilized with ethyl alcohol and sodium hypochlorite, and sown on Murashige and Skoog (MS) medium. Seeds were stratified at 4 °C for 48 h, then transferred to light conditions (21 °C, 16/8 h light/dark cycle). The *N. benthamiana* seedlings were grown in growth chambers at 28 °C under a 12 h light/12 h dark cycle.

### 4.2. Isolation and Functional Characterization of the GhPAP1D Promoter

Genomic DNA was extracted from *R1* cotton and *GL* cotton using the Super Plant Genomic DNA kit (Polysaccharides and Polyphenolics-rich, TIANGEN, Beijing, China). The upstream promoters of *GhPAP1D* in *R1* and *GL* cottons (pro*GhPAP1D^GL^* and pro*GhPAP1D^R1^*) were amplified using PrimeSTAR^®^ G × L DNA Polymerase kit (Takara, Beijing, China), and the primers used for amplifying the promoters are listed in Supplemental Table S2. Analysis of the promoter regions was performed using the database Plantcare (https://bioinformatics.psb.ugent.be/webtools/plantcare/html/, accessed on 4 October 2019) [15].

### 4.3. Stable Transformation in A. thaliana and GUS Staining Assays

To generate the pro*GhPAP1D^GL^*:*GUS* and pro*GhPAP1D^R1^*:*GUS* constructs, the *GhPAP1D* promoters from *GL* and *R1* cottons were amplified and fused with the GUS coding sequence (CDS), with 35S:*GUS* as the control. For overexpression, the full-length *GhBBX21* and *GhBBX24* CDS were cloned into the pBI121 vector. All constructs were then transformed into *Agrobacterium tumefaciens* strain GV3101. Stable transformation in *A. thaliana* (WT) was performed by the floral-dip method [37]. In brief, inoculate *A. tumefaciens GV3101* containing the recombinant plasmid into LB liquid medium with triple antibiotics (Kan/Gen/Rif). Grow overnight at 28 °C, 200 rpm until OD_600_ = 1.0. Centrifuge, discard supernatant, and resuspend the pellet in infiltration buffer (100 mL 1/2 MS, 5 g sucrose, 20 μL Silwet L-77, 10 μL 1M AS, pH 5.8). Incubate in the dark at room temperature for 2 h. Submerge inflorescences of *A. thaliana* (WT) plants in the suspension for 45 s. After infiltration, incubate plants in the dark for 24 h, then transfer to normal growth conditions. T_0_ seeds were collected and screened on MS medium containing 3% sucrose at 21 °C with 50 μg·L^−1^ kanamycin. After two weeks of selection, kanamycin-resistant seedlings were transplanted to a growth chamber at 21 °C under a 16 h light/8 h dark cycle. Transgenic plants were validated by RT-qPCR.

### 4.4. GUS Staining Assays

Homozygous T_3_ *A. thaliana* transgenic lines from both pro*GhPAP1D^GL^:GUS* and pro*GhPAP1D^R1^:GUS* constructs were used for GUS staining assays. T_3_ transgenic seedlings were incubated in MS medium containing 3% sucrose at 21 °C under a 16 h light/8 h dark cycle or continuous darkness (24 h dark). The 7-day-old seedlings were incubated in GUS staining buffer (Huayueyang) for 24 h at 37 °C. Afterwards, 70% ethanol was used to remove plant pigments, and seedlings were observed using a microscope (Olympus SZX10).

### 4.5. Phylogenetic Analysis of BBX Family Proteins

The protein sequences of *A. thaliana* BBX family were obtained from the TAIR database (https://www.arabidopsis.org/, accessed on 12 October 2019), while those of *G. hirsutum* were retrieved from CottonFGD (https://cottonfgd.org/, accessed on 12 October 2019). Amino acid sequence alignments were performed using Clustal W, and a phylogenetic tree was constructed with MEGA 7.0 using the neighbor-joining (NJ) method with 1000 bootstrap replicates.

### 4.6. Subcellular Localization and Bimolecular Fluorescence Complementation (BiFC) Assays

For Subcellular localization assays, the putative full-length CDS sequences of *GhBBX21*, *GhBBX24*, and *GhHY5* without stop codons were amplified and cloned into the pBI121-GFP vector to construct recombinant constructs (35S:*GhBBX21-GFP*, 35S:*GhBBX24-GFP*, and 35S:*GhGhHY5-GFP*). For BIFC assays, the CDS sequence of *GhHY5* without the terminator was recombined into the pSPYCE-35S vector to construct 35S:*GhHY5-c*, whereas the terminator-removed CDS sequences of *GhBBX21* and *GhBBX24* were inserted into the pSPYNE-35S vector, generating the recombinant constructs 35S:*GhBBX21-n* and 35S:*GhBBX24-n*. All constructs were then transformed into *Agrobacterium* GV3101.

Transient expression assays were performed in *N. benthamiana* leaves using *Agrobacterium*-mediated infiltration [38]. The constructs 35S:*GhBBX21-GFP*, 35S:*GhBBX24-GFP,* and 35S:*GhHY5-GFP* were introduced, with 35S:*GFP* serving as a control. After 48 h of incubation, fluorescence was visualized using a confocal microscope (Leica SP8, Wetzlar, Germany).

The BiFC assays allow the detection and verification of potential protein–protein interactions in vivo [39]. *Agrobacterium* was cultured at 28 °C and suspended in the infiltration buffer (100 mL MS with 10 mM MgCl_2_, 100 μM acetosyringone, and 10 mM MES, pH 5.8) to an OD600 of 1.0. The resuspended fluid of 35S: *GhHY5*-c and 35S: *GhBBX*s-n was mixed in a 1:1 volume ratio, allowed to stand for 2 h, and then injected into the back of *N. benthamiana* leaves. After 48 h of normal light exposure at 28 °C, it was observed using the confocal laser microscope (Leica SP8, Wetzlar, Germany).

### 4.7. Dual-Luciferase Assays

The CDS sequences of *GhPAP1D*, *GhBBX21*, *GhBBX24*, and *GhHY5* were cloned into pGreenII 62-SK to generate the effector constructs, whereas the promoter sequences of *GhPAP1D* were inserted into the multi-cloning site of pGreen0800-LUC to generate the reporter constructs. All constructs were individually transformed into *Agrobacterium* GV3101 (*psoup-p19*). Dual-luciferase assays were performed with *N. benthamiana* leaves as previously reported [40]. The *firefly luciferase* and *Renilla* luciferase activities were analyzed three days after infiltration using the Dual-Luciferase Reporter Assay System (E1910, Promega), according to the manufacturer’s instructions.

### 4.8. Yeast One-Hybrid (Y1H) Assay

Y1H assays were performed using the Matchmaker Gold Yeast one-Hybrid System kit (Takara, Beijing, China) according to the manufacturer’s instructions. In brief, the CDS sequences of *GhBBX21*, *GhBBX24* and *GhHY5* were amplified and inserted into pGADT7 to generate the prey constructs, and the promoter sequence (−625 bp to −1 bp) of *GhPAP1D* in *R1* cotton was amplified and inserted into pAbAi to generate the bait construct. The recombinant bait construct pAbAi-pro*GhPAP1D^R1^* was then linearized and introduced into Y1HGold yeast strain cells. The transformed cells were selected on SD/-Ura plates. After determining the minimal inhibitory concentrations of AbA (200 ng·mL^−1^) for the bait construct pAbAi-pro*GhPAP1D^R1^*, the prey constructs were transformed into Y1HGold cells harboring the pAbAi-pro*GhPAP1D^R1^*, and tested on SD/-Leu/AbA plates with an AbA concentration of 200 ng·mL^−1^. The empty vector control was pGADT7 + pAbAi-pro*GhPAP1D^R1^*. The positive control was pGADT7-rec-53 + pAbAi-P53, and the negative control was pGADT7 + pAbAi-P53.

### 4.9. Anthocyanin Content Analysis

Three independent homozygous T_3_ *A. thaliana* transgenic lines from each of the 35S:*GhBBX21* and 35S:*GhBBX24* constructs were used for anthocyanin content assays, with *A. thaliana* (WT) as a control. Anthocyanin content was measured following a published method [41] with minor modifications. Briefly, T_3_ transgenic seedlings were incubated in MS medium containing 3% sucrose at 21 °C under a 16 h light/8 h dark cycle. The 7-day-old *A. thaliana* seedlings were flash-frozen in liquid nitrogen and homogenized to a fine powder. The extraction was performed using a solution of 40% methanol, 10% acetic acid, and 50% ddH_2_O (*v*/*v*). After 4 h incubation at 4 °C in darkness, samples were centrifuged at 8000 rpm for 10 min. The supernatant absorbance was measured at 530 nm and 657 nm using a UV-Vis spectrophotometer (Persee T9, Beijing, China). Anthocyanin content was calculated as (A_530_—0.25 × A_657_) per gram fresh weight.

## 5. Conclusions

Our findings indicate that GhHY5, GhBBX21, and GhBBX24 mediate anthocyanin biosynthesis in *R1* cotton. GhBBX24 binds to the *GhPAP1D* promoter, suppressing its expression and inhibiting anthocyanin biosynthesis. GhHY5 blocks GhBBX24 from binding to the *GhPAP1D* promoter. Meanwhile, GhBBX21 binds to the *GhPAP1D* promoter, leading to a marked upregulation of *GhPAP1D* expression. Notably, compared to *A. thaliana*, the members of the BBX IV subfamily are significantly expanded in *G. hirsutum*. We have identified, for the first time, two BBX IV subfamily members, GhBBX21 and GhBBX24, as regulators of anthocyanin biosynthesis in cotton. However, the mechanisms by which other BBX members in this subfamily regulate anthocyanin accumulation require further investigation.

## Data Availability

The data presented in this study are available on request from the corresponding author.

## Supplementary Materials

The following supporting information can be downloaded at https://www.mdpi.com/article/10.3390/plants14152367/s1, Table S1: primers used in this paper; Table S2: the promoters of *GhPAP1D* in *GL* and *R1* cottons; Figure S1: the autoregulation activities of *GhPAP1D* in *GL* and *R1* cottons (note: effects of GhPAP1D in combination on pro*GhPAP1D^GL^* and pro*GhPAP1D^R1^*, the 62SK as control); Figure S2: the sequence alignment and bZIP domain analysis of AtHY5 and GhHY5; Figure S3: the protein sequences alignment and structural analysisof BBX family Subgroup IV.
