# Supplementary material for: Two B-Box Proteins, GhBBX21 and GhBBX24, Antagonistically Modulate Anthocyanin Biosynthesis in R1 Cotton
Source: Plants (Basel). 2025 Aug 1;14(15):2367. doi: 10.3390/plants14152367 (PMC12348744; doi:10.3390/plants14152367)
Supplement: Supplementary file 1 [file plants-14-02367-s001.zip › plants-3740677-supplementary.pdf]

Supplementary

Table S1 The promoters of *GhPAP1D* in *GL* and *R1* cottons

promoter name	promoter sequences (5'-3')
pro*GhPAP1D*^{GL}	CATGCTATCAAAATGACTAAATATTATTTTTACTATCATAAAAATATATAATTTAATTCCGCCTAACTCAACC<br>TCTGCCATTCATGTAAGCTACGTCACATTGAATAATACAAATATTTGTACGTAGGCAAAGTTGAAATGTTT<br>TGGAATTATATAGTTGGAATGGTAGCTGCAATCAATACTTTTTAAAACAGAGCCGTCACTACTTACTTTCT<br>CTTAGGAGGAACTTTTCTGCGGATAAGAAACCATGCACGTCGCCATTGTTATTAAGGCTTTTCTATCTCC<br>CCCTGAGATTCTAAGCTATAAATAAACGGAGGTTGGCCAACACAGGACAGTATAGAAAAAGTGACATTT<br>TATAAACACACCAAGCTAGCAAGCTAACTATAACAGCTTTTATAAATCGT
pro*GhPAP1D*^{R1}	CATGCTATCAAAATGACTAAATATTATTTTTACTATCATAAAAATATATAATTTAATTCCGCCTAACTCAACC<br>TCTGCCATTCATGTAAGCTACGTCACATTGAATAATACAAATATTTGTACGTAGGCAAAGTTGAAATGTTT<br>TGGAATTATATAGTTGGAATGGTAGCTGCAATCAATACTTTTTAAAACAGAGCCGTCACTACTTACTTTCT<br>CTTAGGAGGAACTTTTCTGCGGATAAGAAACCATGCACGTCGCCATTGTTTTAAAAATATATAATTTAATT<br>CCGCCTAACTCAACCTCTGCCATTCATGTAAGCTACGTCACATTGAATAATACAAATATTTGTACGTAGGC<br>AAAGTTGAAATGTTTTGGAATTATATAGTTGGAATGGTAGCTGCAATCAATACTTTTTAAAACAGAGCCG<br>TCACTACTTACTTTCTCTTAGGAGGAACTTTTCTGCGGATAAGAAACCATGCACGTCGCCATTGTTATTA<br>AGGCTTTTCTATCTCCCCCTGAGATTCTAAGCTATAAATAAACGGAGGTTGGCCAACACAGGACAGTATA<br>GAAAAAGTGACATTTTATAAACACACCAAGCTAGCAAGCTAACTATAACAGCTTTTATAAATCGT

Table S2 Primers were used in this paper.

assay	primer name	primer sequences (5'-3')
Over expression	Over-GhBBX21F	GAGAACACGGGGGACTCTAGAATGAAGATCCAGTGCGATGTCTG
	Over-GhBBX21R	GACCACCCGGGGATCCCCAGAGACGCTTAGATCTCTTGG
	Over-GhBBX24F	GAGAACACGGGGGACTCTAGAATGAAAATCCAGTGTGATGTTTGTGAG
	Over-GhBBX24R	GACCACCCGGGGATCCTCAACCTAGATCAGGGACTGTGAAG
Subcellular localization	GFP-GhBBX21F	GAGAACACGGGGGACTCTAGAATGAAGATCCAGTGCGATGTCTG
	GFP-GhBBX21R	TCATACTAGTCCCGGGGATCCACCAGAGACGCTTAGATCTCTTGG
	GFP-GhBBX24F	GAGAACACGGGGGACTCTAGAATGAAAATCCAGTGTGATGTTTGTGAG
	GFP-GhBBX24R	TCATACTAGTCCCGGGGATCCAACCTAGATCAGGGACTGTGAAG
	GFP-GhHY5F	GAGAACACGGGGGACTCTAGAATGCAAGAACAAGGAACGAG
	GFP-GhHY5R	TCATACTAGTCCCGGGGATCCAAAGGGTTCCATCAGCTGC
Dual-luciferase assay	LUCproGhPAP1D-F	aggtcgacggtatcgataagcttCATGCTATCAAAATGACTAAATATT
	LUCproGhPAP1D-R	ccgctctagaactagtggatccACGATTTATAAAAGCTGTTATAGTTA
	SK-GhHY5F	ccgctctagaactagtggatccATGCAAGAACAAGGAACGAG
	SK-GhHY5R	aggtcgacggtatcgataagcttTTAAAGGGTTCCATCAGCTGC
	SK-GhBBX21F	cgctctagaactagtggatccATGAAGATCCAGTGCGATGTCTG
	SK-GhBBX21R	gtcgacggtatcgataagcttCTACCAGAGACGCTTAGATCTCTTGG
	SK-GhBBX24F	cgctctagaactagtggatccATGAAAATCCAGTGTGATGTTTGTGAG
	SK-GhBBX24R	gtcgacggtatcgataagcttTCAACCTAGATCAGGGACTGTGAAG
	SK-GhPAP1DF	ccgctctagaactagtggatccATGGAAGGCTCATCTTTAAGAGTTAG
	SK-GhPAP1DR	aggtcgacggtatcgataagcttCTATGGGTTGAACACATTCCACA
Promoter cloning	proGhPAP1DF	CATGCTATCAAAATGACTAAATATT

	proGhPAP1DR	ACGATTTATAAAAGCTGTTATAGTTA
BIFC	GhBX21F-BIFC	GAGAACACGGGGGACTCTAGAATGAAGATCCAGTGCGATGTCTG
	GhBX21R-BIFC	GACAGTACTATCGATGGATCCCCAGAGACGCTTAGATCTCTTGG
	GhBX24F-BIFC	GAGAACACGGGGGACTCTAGAATGAAAATCCAGTGTGATGTTTGTGAG
	GhBX24R-BIFC	GACAGTACTATCGATGGATCCACCTAGATCAGGGACTGTGAAG
	GhHY5F-BIFC	GAGAACACGGGGGACTCTAGAATGCAAGAACAAGGAACGAG
	GhHY5R-BIFC	GACAGTACTATCGATGGATCCAAGGGTTCCATCAGCTGC
Gus staining	GUS-proGhPAP1DF	CATGATTACGCCAAGCTTCATGCTATCAAAATGACTAAATATT
	GUS-proGhPAP1DR	GACCACCCGGGGATCCACGATTTATAAAAGCTGTTATAGTTA
Yeast one-hybrid assay	pAbAi632F2413	aattgaaaagcttgaattcgagctcCATGCTATCAAAATGACTAAATATT
	pAbAi632R2413	atacatacagagcacatgcctcgagACGATTTATAAAAGCTGTTATAGTTA
	ADHY5F	GCCATGGAGGCCAGTGAATTCATGCCGCCGAAGAGAAA
	ADHY5R	CAGCTCGAGCTCGATGGATCCTTATCTTCGCATTACACGGTTACG
	ADGhBX21F	GCCATGGAGGCCAGTGAATTCATGAAGATCCAGTGCGATGTCTG
	ADGhBX21R	CAGCTCGAGCTCGATGGATCCCTACCAGAGACGCTTAGATCTCTTGG
	ADGhBX24F	GCCATGGAGGCCAGTGAATTCATGAAAATCCAGTGTGATGTTTGTGAG
	ADGhBX24R	CAGCTCGAGCTCGATGGATCCTCAACCTAGATCAGGGACTGTGAAG

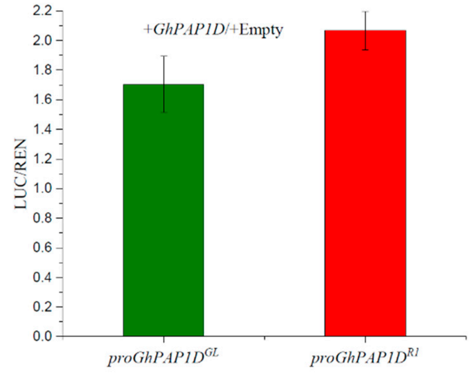

Figure S1 The autoregulation activities of *GhPAP1D* in *GL* and *R1* cottons (note: effects of GhPAP1D in combination on pro*GhPAP1D*^{GL} and pro*GhPAP1D*^{R1}. *Firefly luciferase* activity was normalized to *Renilla luciferase* activity. Error bars represent the mean ± SE of four biological replicates. Significant differences were determined by Student's t-test (*P<0.05, **P<0.01, ***P<0.001, vs empty vector control).

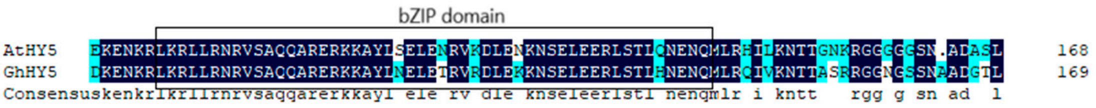

Figure S2 The sequence alignment and bZIP domain analysis of AtHY5 and GhHY5

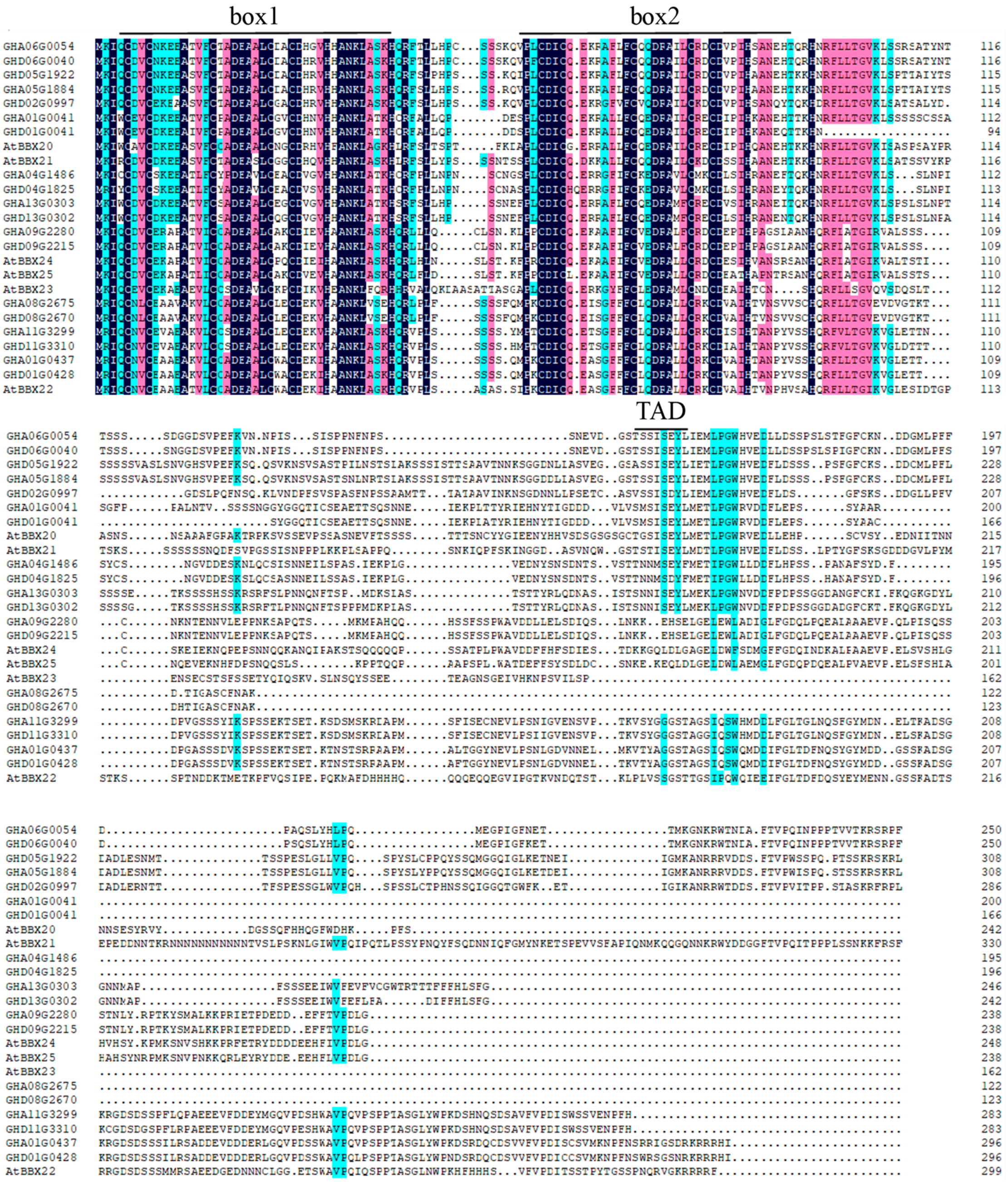

Figure S3 The protein sequences alignment and structural analysisof BBX family Subgroup IV.
